# Analysis of clinical characteristics, treatment patterns, and outcome of patients with bilateral testicular germ cell tumors

**DOI:** 10.1007/s12672-024-00874-9

**Published:** 2024-02-07

**Authors:** Hoffman Azik, Nativ Omri, Malshy Kamil, Haifler Miki, Golan Shay, Mano Roy, Freifeld Yuval, Rosenzweig Barak, Shalom Ben, Stabholz Yariv, Ben-David Reuven, Amiel E. Gilad

**Affiliations:** 1 Urology Department, Rambam Health Care Campus, Haifa, Israel; 2 Israeli Uro-Oncology Consortium, Tel Aviv, Israel; 3 Urology Department, Rabin Medical Center, Pethach Tikva, Israel; 4 Urology Department, Sourasky Medical Center, Tel Aviv, Israel; 5 Urology Department, Carmel Medical Center, Haifa, Israel; 6 Urology Department, Sheba Medical Center, Ramat Gan, Israel

**Keywords:** Testicular cancer, Bilateral germ cell neoplasia, Metachronous, Synchronous

## Abstract

**Introduction:**

Bilateral testicular germ cell tumor (BGCT) is a rare disease, occasionally considered to be more aggressive than unilateral germ cell tumors (GCT) in some reports. Among BGCT, a synchronous disease might be diagnosed at a higher stage than a metachronous disease, resulting in lower cancer-specific survival. Hence, our study aimed to perform a comparative analysis between unilateral testicular GCT, bilateral synchronous GCT, and bilateral metachronous GCT, aiming to verify the possibility that BGCT is diagnosed with a higher stage and may require more aggressive management.

**Material and methods:**

In our multicenter retrospective study we reviewed medical records of 40 patients with BGCT (24 metachronous and 16 synchronous). Clinical characteristics, pathological features of the primary and secondary tumors, adjuvant treatments (chemotherapy and radiotherapy)and sperm quality were evaluated as well as cancer-specific survival and overall survival. A cohort of one-to-one matched patients with unilateral GCT were used to determine risk factors for developing BGCT.

**Results:**

Patients with BGCT were slightly younger compared to those with unilateral GCT and had more advanced disease. Despite similar T-stage distribution between the two groups, nodal involvement was nearly twofold more frequent in patients with BGCT disease (42% vs 22%, p = 0.056). Additionally, although similar histological subtypes distribution at presentation among the two groups, the synchronous disease was diagnosed with a higher local T-stage (OR = 3.4), higher proportions of patients with elevated serum BHCG levels, and more frequent nodal involvement (OR = 2.2). This was later translated into an 18% higher disease-specific mortality rate. The median time to develop a contralateral tumor was 92 months. Pathological local T-stage (T2–T3) of the primary tumor predicted a shorter time interval to a diagnosis of a second contralateral tumor (HR 0.92, P < 0.05).

**Conclusion:**

BGCT presents at a younger age and potentially with more advanced disease. Synchronous BGCT is diagnosed at a later stage compared to metachronous BGCT and has higher disease-specific mortality. Metachronous tumors might have a long time interval for the development of a contralateral neoplasm. The main predictor of developing an early metachronous disease is a high primary T stage.

## Introduction

Testicular cancer incidence is estimated at 1–1.5% among men in the Western world, and it is the most common malignancy in men aged 15–35 [1]. The major histological subtype of testicular cancer consists of germ cell tumors (GCT), 60% of which are non- seminoma and the remaining are seminoma. Over the last 40 years, advances in imaging and treatment modalities have made this cancer a highly curable disease [2]. Among germ cell tumors, bilateral testicular GCT is rare, reported in only 3–5% of GCT patients. Mrinakova et al [3] reviewed a cohort of 2124 GCT patients and reported 4.5 % of bilateral germ cell tumors, mostly metachronous. The median time to develop a secondary tumor was 8.2 years. Approximately 35% of these men present with synchronous tumors (mostly seminoma), and 65% present with metachronous tumors, usually with different pathology in each testicle [4]. Known risk factors for developing Bilateral germ cell tumor (BGCT) include cryptorchidism, Klinefelter’s syndrome, first-degree relative diagnosed with GCT, secondary infertility, and atrophic testicle [5–7]. Additionally, the relative risk of a second contralateral metachronous tumor can reach up to 27 times higher in men diagnosed with a germ cell tumor compared to the general population [6]. In the US, the 5 year cancer-specific survival rate of unilateral testicular cancer is over 95% [8]. However, it has been shown that synchronous BGCT is more aggressive than metachronous disease and results in lower overall survival and cancer-specific survival [9, 10].

## Objectives

To perform a comparative analysis between unilateral testicular GCT, synchronous BGCT, and metachronous BGCT. This is to verify the possibility that a synchronous BGCT disease is indeed a diverse form that may require more aggressive management.

## Materials and methods

### Data acquisition

Using the Israeli National Cancer Registry, we identified 40 patients from six different medical centers, diagnosed with BGCT between the years 2000–2020. The patient's medical records were reviewed for clinical characteristics, pathological features of the primary and secondary tumors as well as adjuvant treatments (chemotherapy and radiotherapy), and sperm quality. Cancer-specific survival and overall survival were obtained using the national population registry. Data collection and analysis was conducted under relevant guidelines and regulations and was approved by all Institutional Review Board (0682-18-RMC) of participating centers.

### Outcome measurements and statistical analysis

To determine risk factors for BGCT, we compared clinical data to a cohort of 40 consecutive unilateral GCT patients matched on-to-one according to time since initial referral to a urologist until initial orchiectomy. In patients with synchronous BGCT, the primary tumor was determined as the larger lesion based on the post-orchiectomy pathology report. Initially, a comparison was made between unilateral GCT and BGCT to evaluate clinical and pathological differences. Independent risk factors for developing BGCT were determined by univariant analysis using Fisher Exact and Mann-Whitney U tests for categorical and continuous variables, respectively. Next, we compare synchronous and metachronous BGCT using similar tests. Multivariable Cox regression analysis was performed to detect independent predictive variables for BGCT. Statistical analysis was performed using SPSS© 21.0 software for Windows.

## Results

We identified 40 patients diagnosed with BGCT, of which 16 had synchronous and 24 metachronous diseases. BGCT patients were compared to a matched cohort of 40 unilateral GCT patients (Table 1). Patients diagnosed with BGCT were slightly younger compared to those with unilateral GCT (29.2 years vs 31.6 years respectively, P<0.05). A history of previous urological pathology (hypospadias, cryptorchidism, UPJ stenosis, ureteric reflux, etc.) was associated with an increased risk for developing unilateral GCT (OR=2.6, 95%CI 1.1–9.3, P<0.05). Serum tumor markers (AFP, LDH, or BHCG) at primary diagnosis were increased by 2.5-fold in patients with BGCT (OR=1.8, 95%CI 1.2–2.7, p<0.05). Lymph node involvement was more frequent in patients with BGCT compared with those with unilateral GCT (42% vs 22%), nearly reaching statistical significance (p=0.056). All other studied variables were equally distributed among both groups. Disease-specific survival did not differ compared to unilateral GCT.

**Table 1:** Unilateral vs. Bilateral germ cell tumor characteristics

		Unilateral GCT	Bilateral GCT	
No		40	40	
Age at diagnosis (year)		31.6 ± 8.8	29.2 ± 7.7	P < 0.05
Cryptorchidism (%)		3 (7%)	1 (2%)	
Hypospadias (%)		1 (2%)	2 (5%)	
Previous urological history		8 (20%)	3 (7%)	P < 0.05
Familial testicular cancer—1st relative (%)		1 (2%)	0	
Familial cancer—1st relative (%)		1 (2%)	2 (5%)	
Abnormal fertility status (%)		4 (10%)	8 (20%)	
Primary pathological stage (%)	T1	28 (70%)	27 (67%)	
	T2	11 (28%)	11 (28%)	
	T3	1 (2%)	2 (5%)	
Primary pathology (%)	Seminoma	26 (65%)	21 (52%)	
	Non-seminoma	14 (35%)	19 (48%)	
Tumor markers at primary diagnosis (%)	S0	31 (78%)	17 (43%)	P < 0.05
	S1-S3	9 (22%)	23 (57%)	
LN-involvement at primary diagnosis	N0	31 (78%)	23 (58%)	
	N1	8 (20%)	15 (38%)	
	N2	0	1 (2%)	
	N3	1 (2%)	1 (2%)	

*LN* lymph nodes

The clinical and pathological characteristics of patients with synchronous vs metachronous BGCT are summarized in Table 2. The most significant differences noted were: Patients with a metachronous disease were slightly younger and had more often a history of cryptorchidism (12% vs 0%). A more advanced stage at diagnosis was observed among those with synchronous BGCT who had a more frequent incidence of higher (T2–T3), local T-stage (OR=3.4, 95%CI 1.3–9.1, p<0.05) and a higher rate of lymph node involvement (OR=2.2 95%CI 1.1–4.4, p<0.05). Considering serum tumor markers, BHCG was 4-fold more elevated in the patients with synchronous BGCT vs. metachronous BGCT (62% vs 16%). It should be noted that patients with synchronous BGCT had a slightly higher incidence of abnormal sperm count at primary tumor diagnosis compared to metachronous BGCT (25% vs. 16%). Multivariant regression including the age at presentation, pathological T-stage, histological subtype, and lymph node status at presentation did not detect any risk factors for developing a contralateral GCT when comparing patients who eventually developed a metachronous BGCT to those with a unilateral GCT.

**Table 2:** Synchronous vs. Metachronous bilateral germ cell tumors

		Synchronous BTC	Metachronous BTC	
Number of patients		16	24	
Age at diagnosis (year)		30.8 ± 7.1	28 ± 8	
Cryptorchidism (%)		0	3 (12%)	
Hypospadias (%)		2 (12%)	0	
Familial Testicular Cancer (%)		1 (6%)	1 (4%)	
First degree relative GCT (%)		1 (6%)	0	
Abnormal fertility (%)		4 (25%)	4 (16%)	
Primary pathological stage (%)	T1	7 (43%)	15 (63%)	P < 0.05
	T2	7 (43%)	4 (16%)	
	T3	2 (13%)	0	
Primary pathology (%)	Seminoma	8 (50%)	13 (54%)	
	Non-seminoma	8 (50%)	11 (45%)	
Tumor markers at primary diagnosis	# of patients (%)	11 (69%)	18 (75%)	
	AFP	5	7	
	BHCG	10	4	
	LDH	14	7	
LN-involvement at primary diagnosis	N0	6 (37%)	17 (70%)	P < 0.05
	N1	10 (62%)	7 (30%)	
	N2	1 (6%)	0	
Adjuvant chemotherapy		11 (69%)	7 (29%)	
Disease specific survival		12/16 (75%)	22 / 24 (92%)	

*GCT* germ cell tumor, *LN* lymph nodes

At the time of diagnosis, the distribution of histological subtypes (seminoma and non-seminoma) in the primary tumor was almost identical between the two studied groups. However, when all 80 testicles were considered, the proportion of seminoma was higher (62.5%). When comparing the primary and secondary histological subtypes, no correlation was observed in the metachronous group. In the synchronous BGCT group, most histological types of contralateral tumor matched the primary histology. Of note, most metachronous contralateral tumors consisted of seminoma (87.5%).

**Fig. 1:**
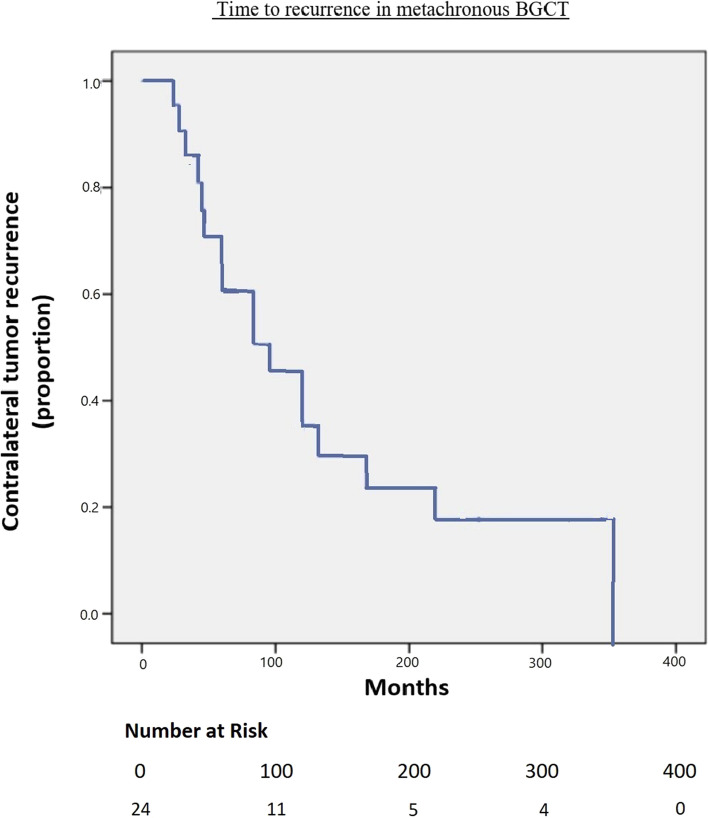
Time-to-recurrence in metachronous BGCT

The time to recurrence in metachronous BGCT is illustrated in Figure 1*.*

The median time to develop a contralateral GCT was 92 months (range 23–350). Only pathological local T-stage (T2–T3) of the primary tumor predicted a shorter time interval to a diagnosis of a second GCT (HR 0.92, P < 0.05). 45% (18/40) of patients with BGCT received chemotherapy and 2.5% (1/40) received radiation. Receiving adjuvant treatment following the initial diagnosis did not affect the time to recurrence in the metachronous BGCT cohort.

Patients with synchronous GCT disease were more likely to receive chemotherapy compared with the metachronous group (69% vs 29%, p<0.05). Six patients (15%) died of GCT following adjuvant treatment during the follow-up period. Four patients were in the synchronous group (25%) and two patients were in the metachronous group (8%). No predictors of disease-specific survival were detected.

## Discussion

There are only a few reported cohorts of BGCT in the literature and no professional-specific guidelines, making it difficult to conclude the optimal management and the required follow-up strategies for this rare disease. The main findings of this current study include the following: Patients with BGCT had more aggressive disease, with nearly 2-fold more frequent nodal involvement compared with those with unilateral disease (42% vs 22%, p=0.056). Despite the equal distribution of histological subtypes at presentation, among the two groups, the synchronous disease was more advanced at diagnosis. This difference was evident by a higher rate of local T-stage (OR = 3.4), higher proportions of patients with elevated serum BHCG levels, and more frequent nodal involvement (OR = 2.2), and was translated into an increased proportion of patients that required chemotherapy and a higher disease-specific mortality rate.

In a systemic review, Campobasso et al [10] reported 33 cases of BGCT consisting of seminoma with an overall survival rate of 100% (median follow-up of 45.4 months), and 11 nonseminoma BGCT, 8 of whom were diagnosed with stage III disease and therefore had 62.5% mortality rate when the disease progressed. They concluded that based on previously reported series, patients with bilateral seminoma presented with a low stage at diagnosis and favorable overall survival, whereas cases with non-seminoma BGCT had a high stage at diagnosis and poor prognosis. The current cohort has different stage distributions. Our current data suggest that synchronous BGCT might involve more aggressive features compared to metachronous disease, therefore treatment decisions regarding adjuvant therapy and the impact on disease-specific survival should be driven by this, regardless of seminoma or non-seminoma on pathology. Decisions regarding adjuvant treatment for synchronous BGCT should still follow current guidelines. However, one should keep in mind that those rare patients are possibly at risk of disease progression.

The median time to diagnosis of a contralateral GCT was nearly 8 years and was associated with the local T-stage at the initial presentation. Those patients with T2 or T3 stage had a shorter interval for detection of BGCT in the contralateral testicle. Naturally, patients diagnosed with unilateral GCT should be submitted to close follow-up initially, allowing early diagnosis of metachronous BGCT, but based on our results, this should be continued for many years.

Previous reports suggest that BGCT consists mainly of seminomas, particularly synchronous BGCT. In one of the largest systematic reviews of BGCT, Zequi et al. describe 91.6% of cases of synchronous tumors presented with seminoma histology [9]. This rate is significantly higher than the 62.5% observed in our study and may explain higher awareness and close follow-up in patients eventually developing metachronous BGCT in our cohort, i.e only 31% of organ-confined disease among synchronous BGCT patients in our study compared with 50% as described by Zequi et al. In contrast, the non-synchronous BGCT group had virtually identical proportions of localized disease in both studies. These differences in histological subtype distribution were ultimately translated into increased disease-specific mortality in our cohort (25% vs. 12%) [9]. Similar findings were also reported by Hentrich et al. who summarized their own experience from 25 years of follow-up. They observed a survival advantage for patients with the metachronous BGCT disease compared with the synchronous BGCT (disease-specific mortality of 3% vs 35%, respectively) [11]. An important finding of our study group was the relatively long-time interval between the diagnosis of primary and secondary tumors in the metachronous BGCT group. In our study, the median duration for diagnosis of a secondary tumor was nearly 8 years. A time interval with a similar magnitude was also reported by Heinrich et al and Zequi et al [9, 11]. The main implication of such finding is the need for long-term and close follow-up of patients with germ cell testicular cancer, especially those with higher T-stage at the time of diagnosis.

Fertility status is a particularly significant concern when treating BGCT. Current recommendations [12] highly support sperm banking for all patients treated for GCT before orchiectomy. Given the increased fertility concern in this unique group of patients, organ-preserving surgery was suggested as a reasonable option in selected patients [13]. Of note, is the relatively high incidence of sperm abnormalities, as up to 25% of patients with synchronous BGCT in our cohort had a pre-orchiectomy abnormal sperm count. Moreover, the relatively high rate of positive lymph node disease in these patients (69%) requires adjuvant radiation therapy or chemotherapy and is expected to cause further fertility impairment.

The current study has several limitations including a small sample size, retrospective nature, lack of central pathology re-evaluation, and slightly different indications for adjuvant treatment in each cancer center. However, the data include a multicenter matched cohort representing the real-life practice in treating BGCT.

## Conclusions

Bilateral germ cell tumors present at a younger age. Synchronous BGCT tends to be diagnosed at a more advanced pathological stage, with higher lymph node involvement and a higher proportion of patients with elevated serum BHCG. These are more often treated with chemotherapy and have higher disease-specific mortality. In metachronous BGCT, we observed a long time interval (92 months) to develop a secondary contralateral tumor. the main predictor to develop the early metachronous disease is a high T-stage at the presentation of the primary tumor. This should be considered when discussing the follow-up protocol with germ cell tumor patients.

## Data Availability

The full database is stored by the corresponding author as instructed by the IRB committee.
